# 1336. Outcomes of COVID-19 in Recent Kidney Transplants Recipients at a Large Transplant Center in Miami

**DOI:** 10.1093/ofid/ofab466.1528

**Published:** 2021-12-04

**Authors:** Maria A Mendoza, Ana Coro, Yoichiro Natori, Shweta Anjan, Giselle Guerra, Adela Mattiazzi, Linda Chen, Mahmoud Morsi, Jose Figueiro, Yehuda Raveh, Jacques Simkins

**Affiliations:** 1 Jackson Memorial Hospital, Miami, Florida; 2 University of Miami, Miami, Florida; 3 Jackson Memorial Hospital/Miami Transplant Institute, University of Miami Miller School of Medicine, Miami, FL; 4 University of Miami / Jackson Memorial Hospital, Miami, Florida

## Abstract

**Background:**

Outcomes of COVID-19 have been reported in deceased donor kidney transplant (DDKT) recipients. However, data is limited in patients that underwent recent DDKT.

**Methods:**

This single-center retrospective study evaluated the differences in demographics and post-transplant outcomes between those who tested positive and negative for Severe Acute Respiratory Syndrome Coronavirus 2 (SARS-CoV-2) by polymerase chain reaction, after undergoing recent DDKT. The treatments and outcomes for the SARS-CoV-2-positive patients were assessed. Patients who underwent DDKT from 3/2020 to 8/2020 were included and followed until 9/2020.

**Results:**

201 DDKT recipients were analyzed [14(7%) SARS-CoV-2-positive and 187(93%) negative]. There was no difference in delayed graft function and biopsy-proven rejection between both groups. The patient survival at the end of the study follow-up was lower among SARS-CoV-2-positive patients (Table 1). The median time from DDKT to COVID-19 diagnosis was 45 (range: 8-90) days; 5(36%) patients required intensive care unit and 4(29%) required mechanical ventilation; steroids were used in all the patients, therapeutic plasma exchange (TPE) and convalescent plasma (CP) in 7(50%) patients each, remdesivir in 6(43%) and tocilizumab in 1(7%); 9(64%) patients recovered, 3(21%) died and two were still requiring mechanical ventilation at the end of the follow-up.

**Conclusion:**

Our cohort demonstrated a lower survival rate among SARS-CoV-2-positive patients, which highlights the vulnerability of the transplant population. Transplant patients must comply with the CDC recommendations to prevent COVID-19.

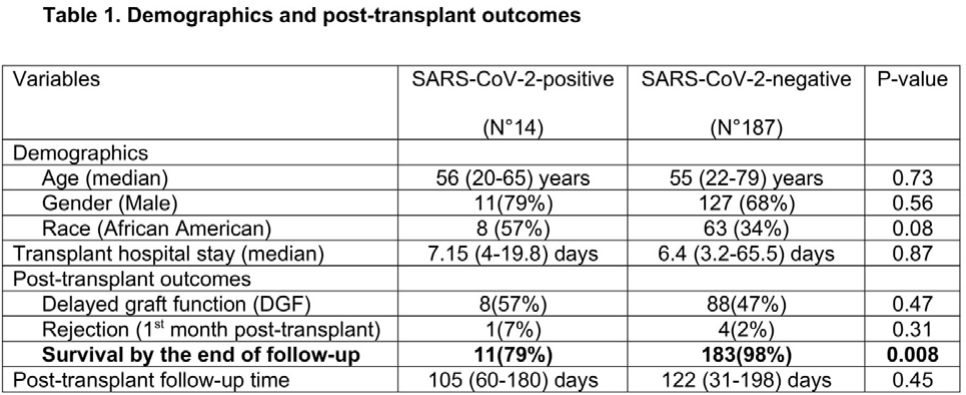

**Disclosures:**

**All Authors**: No reported disclosures

